# Physiological parameters to support attention deficit hyperactivity disorder diagnosis in children: a multiparametric approach

**DOI:** 10.3389/fpsyt.2024.1430797

**Published:** 2024-11-07

**Authors:** Thais Castro Ribeiro, Esther García Pagès, Anna Huguet, Jose A. Alda, Llorenç Badiella, Jordi Aguiló

**Affiliations:** ^1^ CIBER de Bioingeniería, Biomateriales y Nanomedicina (CIBER-BBN), Instituto de Salud Carlos III, Madrid, Spain; ^2^ Department of Microelectronics and Electronic Systems, Autonomous University of Barcelona, Barcelona, Spain; ^3^ Child and Adolescent Mental Health Service, Sant Joan de Déu Terres de Lleida, Lleida, Spain; ^4^ Children and Adolescent Mental Health Research Group, Institut de Recerca Sant Joan de Déu, Esplugues de Llobregat, Spain; ^5^ Child and Adolescent Psychiatry and Psychology Department, Hospital Sant Joan de Déu, Barcelona, Spain; ^6^ Applied Statistics Service, Autonomous University of Barcelona, Barcelona, Spain

**Keywords:** ADHD (attention deficit and hyperactivity disorder), ADHD classification, physiological parameters, multiparametric models, HRV (heart rate variability), EDA (electrodermal activity)

## Abstract

**Introduction:**

Attention deficit hyperactivity disorder (ADHD) is a high-prevalent neurodevelopmental disorder characterized by inattention, impulsivity, and hyperactivity, frequently co-occurring with other psychiatric and medical conditions. Current diagnosis is time-consuming and often delays effective treatment; to date, no valid biomarker has been identified to facilitate this process. Research has linked the core symptoms of ADHD to autonomic dysfunction resulting from impaired arousal modulation, which contributes to physiological abnormalities that may serve as useful biomarkers for the disorder. While recent research has explored alternative objective assessment tools, few have specifically focused on studying ADHD autonomic dysregulation through physiological parameters. This study aimed to design a multiparametric physiological model to support ADHD diagnosis.

**Methods:**

In this observational study we non-invasively analyzed heart rate variability (HRV), electrodermal activity (EDA), respiration, and skin temperature parameters of 69 treatment-naïve ADHD children and 29 typically developing (TD) controls (7-12 years old). To identify the most relevant parameters to discriminate ADHD children from controls, we explored the physiological behavior at baseline and during a sustained attention task and applied a logistic regression procedure.

**Results:**

ADHD children showed increased HRV and lower EDA at baseline. The stress-inducing task elicits higher reactivity for EDA, pulse arrival time (PAT), and respiratory frequency in the ADHD group. The final classification model included 4 physiological parameters and was adjusted by gender and age. A good capacity to discriminate between ADHD children and TD controls was obtained, with an accuracy rate of 85.5% and an AUC of 0.95.

**Discussion:**

Our findings suggest that a multiparametric physiological model constitutes an accurate tool that can be easily employed to support ADHD diagnosis in clinical practice. The discrimination capacity of the model may be analyzed in larger samples to confirm the possibility of generalization.

## 1 Introduction

Attention deficit hyperactivity disorder (ADHD) is a highly prevalent and heterogeneous neurodevelopmental disorder, characterized by a persistent pattern of inattention, hyperactivity, and impulsive behavior, leading to substantial impairment in functioning (1). Estimates suggest a global prevalence of ADHD of 7.6% in children aged 3 to 12 years, being more common in boys, and frequently associated with other psychiatric and medical conditions (2, 3). In addition to these core symptoms, emerging evidence highlights emotional dysregulation (4) and sensory processing difficulties (5) as potential associated features of ADHD.

The physiopathology of ADHD has been linked to cortical imbalances due to a deficit in dopamine signaling, affecting some specific areas of cortex related to executive function (EF). This is relevant to the executive dysfunctional theory (6), which posits that the ADHD symptoms stem from a primary deficit in executive control due to structural, functional and biochemical abnormalities in neural networks. Although there is evidence of EF weaknesses in individuals with ADHD compared to controls, these deficits alone are insufficient to account for all cases of ADHD (7). Therefore, a different approach has gained significant research interest, the state-regulation theory (8), which emphasizes neurophysiological autonomic dysregulation as a key contributor to both behavioral and cognitive symptoms in ADHD. This model suggests that a diminished ability to regulate arousal through autonomic function may impair an individual’s capacity to meet cognitive demands and adapt to situational challenges. By integrating state factors such as effort, arousal, and activation, this model provides a more comprehensive perspective on the disorder’s heterogeneity.

Evidence suggests that the neuroanatomy and functioning of children with ADHD can resemble those of neurotypical individuals if symptoms are identified and treated early (9). Nevertheless, clinical diagnosis is often delayed due to a lack of a standardized procedure and limited access to specialized health professionals (10). The current diagnostic process typically relies on psychometric questionnaires, face-to-face interviews, and various clinical assessments to identify the presence of symptoms, which is time-consuming and costly. Furthermore, these methods may be biased by inconsistencies in parent-teacher reporting (11) and the clinician’s interpretation of the heterogenous presentations (12). Thus, there is a critical need for objective instruments that provide quick results for ADHD diagnosis, potentially alleviating the clinicians’ workload.

In recent years, substantial efforts have been made to develop objective assessment and predictive tools, such as various neuropsychological tests (e.g. continuous performance test). Despite these advancements, no specific and clinically valid objective biomarker for ADHD has been identified to date (13, 14). A recent review analyzed studies that propose artificial intelligence techniques, to make faster and more accurate diagnoses (15). Among the diagnostic tools used as a reference, neuroimaging, physiological parameters, psychometric questionnaires, sustained attention tests and activity metrics stand out. Cao et al. (16) pointed to the potential benefits that machine learning techniques can add to the diagnosis process, developing increasingly simple and accurate models. To achieve good discrimination capabilities with these techniques, it is important to use reliable labeled data for model design and training.

In line of state-regulation theory, autonomic dysregulation has been described as a common characteristic of ADHD (17, 18). The autonomic nervous system (ANS) plays a vital role in homeostasis, and its inadequate modulation leads to reduced efficiency in performing tasks that require attention, which affects information processing (19). The inability of the ANS to respond flexibly to moment-to-moment demands may leave an individual more likely to be distracted by irrelevant stimuli and less able to detect relevant stimuli. Some easily measured physiological parameters are considered indexes of ANS functioning, including heart rate variability (HRV), which is vagally-mediated (i.e., parasympathetic pathway) and considered a marker of emotional self-regulation (20), and electrodermal activity (EDA), which is modulated by the sympathetic branch of ANS, being a reliable measure of autonomic arousal (21).

Despite the great research interest in HRV in recent decades, few studies have investigated HRV in children with ADHD, and the findings remain inconsistent. Some studies found higher HRV in patients with ADHD (22–24), while others found no statistical differences (25). Typically, higher HRV is associated with better emotional regulation, which contrasts with ADHD’s hallmark difficulties in this area. This paradoxical finding could be interpreted as abnormal hypo-arousal in ADHD, consistent with the arousal dysregulation theory underlying ADHD (26). Additionally, research investigating HRV changes in response to cognitive or behavioral tasks in ADHD remains limited. For instance, Musser et al. (27) found reduced HRV in ADHD compared to controls during an emotion-related task, while other studies reported no differences during sustained attention tasks (28, 29). Bellato et al. (26) suggested that further studies should examine stress-related changes in heart rate (HR) (i.e., HR reactivity), as this may be a more appropriate indicator of ANS reactivity and social-emotional processing.

Regarding EDA, a baseline reduction has been reported in individuals with ADHD compared to controls, for both its phasic (28, 30, 31), and tonic (32, 33) components. Negrao et al. (29) suggested that unmedicated children significantly increase EDA like the healthy controls during a sustained attention task. Halbe et al. (34) demonstrated that the type of task also seems to have an influence, since children with ADHD presented a higher EDA both as an anticipatory response and during a decision-making task, indicating greater emotional arousal than controls. Du Rietz et al. (35) studied EDA in adults with ADHD and suggested that EDA is context-dependent and appears to have fluctuating behavior, which may account for the variability across studies.

Considering this evidence, further research is needed to explore physiological behaviors in ADHD. Until now, no specific biomarker has been established for the ADHD diagnosis, and relying on a single parameter seems insufficient to capture the disorder’s heterogeneity. The development of novel objective tools for detecting ADHD may provide supplementary information, complementing existing screening methods. A multiparametric approach appears to be more appropriate and could yield more comprehensive information related to ANS functioning, as indicated by a similar study conducted in adults with ADHD and healthy controls (36).

Based on previous evidence linking ADHD symptoms to autonomic dysregulation and deficits in arousal, we hypothesize that a set of resting-state physiological parameters provide an easy and non-invasive measure for distinguishing between ADHD and typically developing (TD) children. This study, therefore, investigated a range of physiological parameters, including HRV, EDA, respiration and skin temperature. A similar approach has been applied in previous studies evaluating acute stress (37, 38) and chronic stress (39), conditions also associated with ANS imbalance.

This study shows the potential of a multiparametric physiological model in discriminating ADHD from TD controls, thereby facilitating a faster diagnosis and enabling earlier treatment implementation. We first identified the most relevant parameters exploring the physiological behavior of children with ADHD in contrast to a control group. Subsequently, we developed a logistic regression model to discriminate between the children’s class labels (ADHD *vs.* typically developing controls) using a multivariate approach.

## 2 Materials and methods

### 2.1 Participants and eligibility criteria

This observational study included two different groups. The first group (ADHD Group) consisted of children recently diagnosed with ADHD, treatment-naïve, recruited at Hospital Sant Joan de Déu (HSJD), in the ADHD Unit, and the Child and Adolescent Mental Health Services of Mollet del Vallés (Barcelona, Spain). This sample was part of a randomized controlled trial to evaluate the effectiveness of a mindfulness-based intervention. While the intervention is beyond the scope of this manuscript, the data analyzed corresponds exclusively to the pre-intervention phase. A clinical interview was conducted by a specialist with the children and their parents to screen for eligibility and confirm their participation.

Inclusion criteria for ADHD group: aged 7-12 years; diagnosis of ADHD by a specialist during the 3 months before the study according to Diagnostic and Statistical Manual of Mental Disorders (DSM-5) classification; score greater than 1.5 standard deviations (SD) of normality for corresponding age and gender on the ADHD Rating Scale-IV (ADHD-RS-IV) parents’ version. Exclusion criteria: intelligence quotient (IQ) below 70; diagnosis of autism spectrum disorder or bipolar disorder; being medicated.

The second group (TD group) was formed by typically developing (TD) children recruited at the Hamelin-Laie International School (Barcelona, Spain) as an extension of the above-mentioned ADHD study.

Inclusion criteria for TD group: aged 7-12; absence of diagnosis of ADHD or other neurodevelopmental disorders. Exclusion criteria: IQ below 70; diagnosis of mental disorder.

### 2.2 Experimental procedure

After the approval of the Ethics Committee of the HSJD (PIC-187-15), the ADHD group was recruited between July 2016 and January 2019. Similarly, when the Research Committee of the Autonomous University of Barcelona (UAB) approved the study extension (CEEAH 4936), the TD controls were recruited between February and March 2020. Written informed consent was obtained from all the parents or legal guardians of the participants.

**Figure 1:**
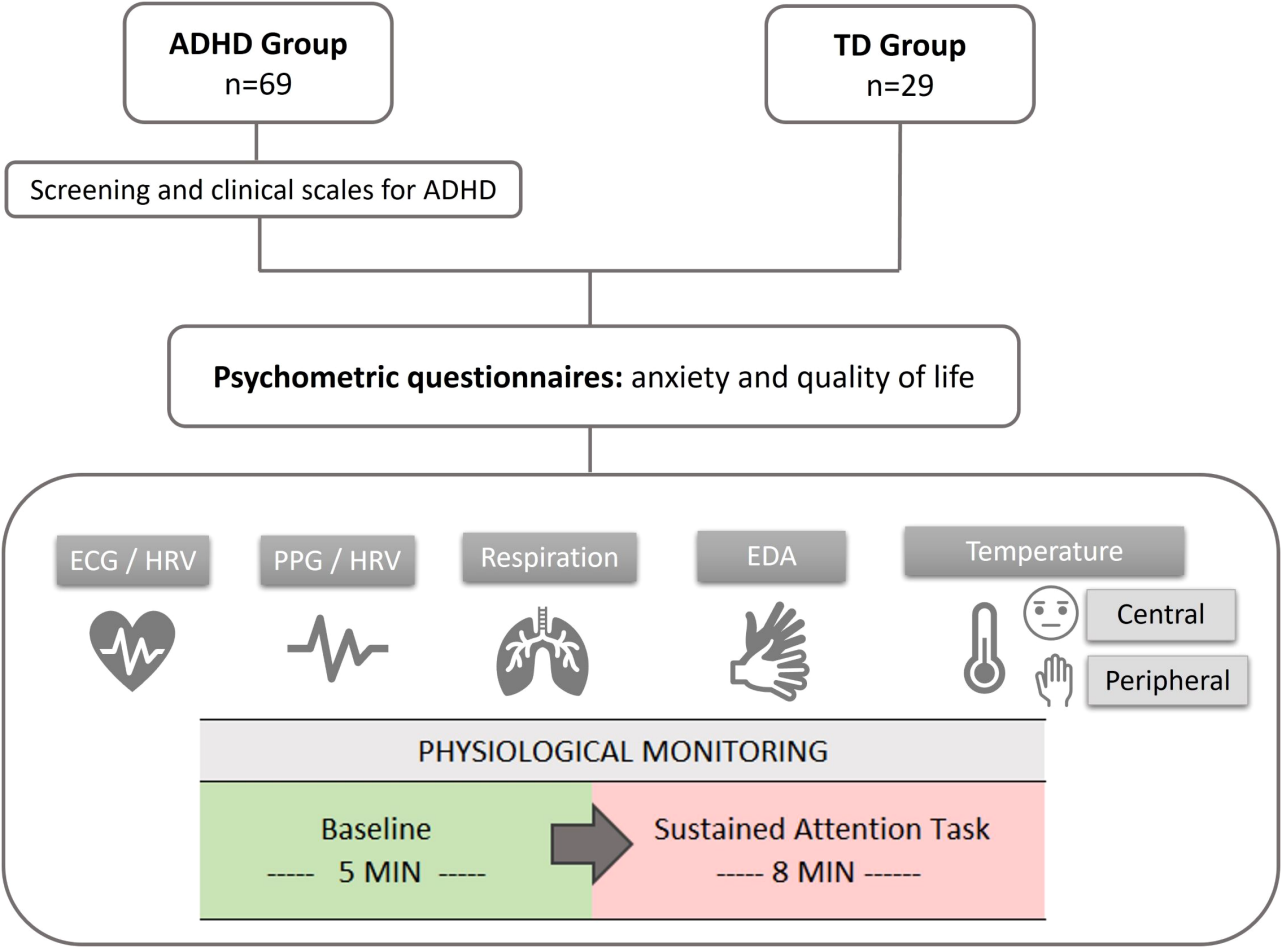
Scheme of the experimental procedure. ECG, electrocardiogram; HRV, heart rate variability; PPG, photoplethysmography; PRV, pulse rate variability; EDA, electrodermal activity.

Firstly, the children auto filled some psychometric questionnaires, and, subsequently, physiological monitoring was carried out. The monitoring session was divided into two parts: (a) Baseline stage: in a resting state with the eyes-open; and (b) Stress stage: during a computer-based sustained attention task (see **Figure 1**).

The specific task used differed between groups due to logistical reasons. The ADHD group completed the CPT-3 (Conners Continuous Performance Task, 3rd Edition) (40), while the TD group performed the CSAT-R (Children Sustained Attention Task - Revised) (41). Both tasks were designed to assess attention performance in children. To minimize the impact of using different tasks, the model described in subsection 3.3 was developed using only the physiological data collected during the baseline stage.

### 2.3 Measures

#### 2.3.1 Psychometric and clinical questionnaires

- Anxiety: assessed with the Screen for Child Anxiety Related Disorders (SCARED) (42, 43). The instrument consists of 41 items and is designed to evaluate anxiety symptoms in children and young people. A total score >30 indicates the presence of clinical symptoms. An individual total score can also be obtained for each domain.
- Quality of life: evaluated with the Child Health an Illness Profile (CHIP) Child Report Form (44, 45). It contains 44 items and evaluates the perceived health-related quality of life in children. The items are distributed in 5 domains: satisfaction, comfort, resilience, risk avoidance, and achievement. Higher scores indicate better perceived health.

To describe the ADHD group, we used 2 clinical scales:

- Intensity of ADHD symptoms: the parent version of the ADHD-RS-IV (46) was used. This instrument includes 18 items that evaluate the diagnostic criteria of ADHD according to the DSM-IV. The higher the scores on the scales, the more intense the presence of the symptoms. It is divided into 3 subscales: inattention, hyperactivity-impulsivity, and total symptoms. The internal consistency of the Spanish version is good (α = 0.86) (47).
- Emotional dysregulation (DESR): deficient emotional self-regulation refers to a theoretical concept characterized by poor modulation of emotional responses, including symptoms such as mood lability, impulsivity and temper outbursts. DESR was assessed through the Child Behavior Checklist (CBCL) scale (48). The emotional dysregulation profile (49, 50) is calculated by the sum of the standardized scores of anxiety/depression, attention problems, and aggressive behavior scales. PT ≤ 179 indicates adequate emotional regulation; PT≥180 and <210 indicate mild-moderate emotional dysregulation; PT ≥ 210 points to severe dysregulation.

#### 2.3.2 Physiological parameters

All physiological signals were simultaneously recorded through a medical grade device: Medicom 83 system, ABP-10 module (Medicom MTD Ltd). The raw signals were analyzed in 1-minute windows using the BioSigBrowser (51) in MATLAB software v.2018a (The MathWorks Inc), and different parameters were extracted (see **Table 1**). The signal conditioning and variable extraction followed the procedure described in (37), which was previously used for stress assessment in healthy students.

**Table 1:** Description of extracted parameters from electrophysiological signals.

Physiological signal	Sample frequency	Extracted parameters	Description
ECG^a^ for HRV^b^ or PPG^c^ for PRV^d^	1000 Hz	HR^h^, bpm	Mean HR
		SDNN, s	SD^i^ of normal beats intervals
		RMSSD, s	Root mean square of successive differences between beat intervals
		PLF, s^-2^	Absolute power in LF^j^ band (0.04-0.15 Hz)
		PHF, s^-2^	Absolute power in HF^k^ band (0.15-0.4 Hz)
		LF/HF, AD^l^	Ratio of LF to HF power
		PHFn, nu	Relative power of the normalized HF band
ECG and PPG	1000 Hz	PAT^m^, ms	Mean PAT, the time between the beat detected by ECG and the pulse by PPG
		stdPAT, ms	SD of PAT
Resp^e^	256 Hz	RR^n^, Hz	Mean RR
		Pk, %	Peak of the respiratory power spectrum
EDA^f^	256 Hz	Tonic, µS	Average value of the tonic component, i.e. slowly changing level, also known as SCL°
		stdTonic, µS	SD of the tonic component
		Phasic, µS	Average value of the phasic component, i.e. fast-changing responses typically associated with short-term events, also known as SCR^p^
		stdPhasic, µS	SD of the phasic component
		aucPhasic, µS·s	Area under the curve of the phasic component
		EDASymp, AD	Electrodermal response in the power spectrum of 0.045-0.25 Hz
ST^g^	256 Hz	TFinger, °C	Mean finger temperature
		TFace, °C	Mean face temperature
		TGrad, °C	Average of successive ST differences every 10 s
		TPow, °C^2^	Mean power of temperature
		TRatio, AD	Ratio between peripheral (finger) and proximal (face) temperature

^a^ECG, electrocardiogram; ^b^HRV, heart rate variability; ^c^PPG, photoplethysmography; ^d^PRV, pulse rate variability; ^e^Resp, respiration; ^f^EDA, electrodermal activity; ^g^ST, skin temperature; ^h^HR, heart rate; ^i^SD, standard deviation; ^j^LF, low frequency; ^k^HF, high frequency; ^l^AD, adimensional; ^m^PAT, pulse arrival time; ^n^RR, respiratory rate; °SCL, Skin Conductance Level; ^p^SCR, Skin Conductance Responses.

To process the electrocardiogram (ECG), beat detection was performed using a discrete wavelet transform (52). Subsequently, ectopic beats or false QRS detections were corrected before computing the interbeat interval series (53). Heart rate variability (HRV) parameters were then calculated using both time-domain and frequency-domain analysis by Fourier transform of the instantaneous heart rate signal. For the photoplethysmography (PPG), artifacts were suppressed using a Hjorth parameter-based PPG artifact detector (54). Pulses were detected in artifact-free time windows using an algorithm reported by Lázaro et al. (55). The same ECG parameters are also extracted in PPG as pulse rate variability (PRV). The mean time difference between the R peak in the ECG signal and the point of 50% increase, corresponding to the pulse detected on the finger by the PPG signal, was computed as the pulse arrival time (PAT).

From the respiration signal, the respiratory rate was estimated as the frequency corresponding to the maximum peak of the power density spectrum (56). If the spectral peak was greater than 65%, the time window was considered stable and valid. The EDA signal is visually inspected to remove motion artifacts and linearly interpolated. A time-domain analysis was performed using a convex optimization model, called cvxEDA, to calculate the tonic and phasic components (57). A frequency-domain analysis was also conducted to assess sympathetic tone through a parameter named EDASymp (58). Finally, for the skin temperature (ST) signal, visual inspection was performed to identify and discard segments with large artifacts before calculating the parameters.

HRV is also analyzed using an extended band, i.e. the upper limit of the high-frequency (HF) band adjusted at half the average heart rate, as described in (59). This approach aims to avoid over or under-estimating parasympathetic activity (related to the power in HF).

### 2.4 Statistical analysis

We conducted the statistical analysis using SAS v.9.4 (SAS Institute). A 95% confidence interval was assumed, and the significance level was 0.05. A descriptive analysis was performed to summarize the children’s characteristics of the sample by groups. Continuous variables are presented as mean and standard deviation (SD), and categorical variables are presented as frequency and percentages. To determine the association or independence between categorical variables, the Chi-square test (χ²) was used. To contrast continuous variables, the Student T-test or the Mann-Whitney U test were used depending on the data distribution. The normality of the distribution was determined using the Shapiro-Wilk test. Skewed variables were analyzed using a log transformation.

To design the classification model, we performed a multiple imputation (by Z scores) for missing data of physiological parameters using the multivariate Maximum Likelihood method. The imbalance between study groups regarding age and gender was compensated with a propensity score matching (PSM) technique using the logarithm of the propensity scores and a greedy nearest neighbor method to match observations (60). For variable reduction, the variance inflation factor (VIF) was the criterion applied. A high VIF value indicates multicollinearity, i.e. a certain parameter is already explained by other parameters, showing a high correlation. Thus, following this approach, according to VIF estimates and the reviewed literature, the parameters that provide redundant information were eliminated.

Finally, we explored physiological parameters as explanatory variables of the class label (ADHD *vs.* TD) using a logistic regression model. The model was adjusted by age and gender. Moreover, a backward stepwise selection method was used to obtain a simplified model (criterion *p* < 0.05). We also explored the presence of interactions between explanatory variables, but none of them were found relevant. The area under the ROC curve (AUC) assessed the model’s performance. All physiological data was previously standardized so that the model’s coefficients could be more easily interpreted. Generalized linear models were used to verify the relationship between model-predicted scores and other moderating effects.

## 3 Results

In total, 98 children were included in the study, 69 ADHD and 29 TD controls. All sample characteristics are detailed in **Table 2**. The TD group was predominantly formed by girls (62.1%) with a mean age of 9.14 years. On the other hand, the ADHD group was formed mostly by boys (73.9%) with a mean age of 8.95 years. A significant difference between groups was found for gender (*p* = 0.001).

**Table 2:** Sociodemographic and psychometric characteristics of the sample.

Variable	TD group (n=29)	ADHD group (n=69)	*p*-value (test)
	Mean (SD)		
**Age**	9.14 (0.92)	8.95 (1.51)	0.360 (A)
**Quality of life (CHIP)** Satisfaction Comfort Resilience Risk avoidance Achievement	46.09 (11.62) 42.67 (10.50) 43.88 (13.40) 51.15 (9.57) 54.04 (9.57)	43.49 (13.80) 45.29 (11.75) 42.32 (11.64) 39.70 (13.39) 36.07 (11.44)	0.491 (A) 0.187 (A) 0.565 (B) <0.001 (A)* <0.001 (B)*
**Anxiety (SCARED)**	24.31 (7.92)	25.01 (12.41)	0.723 (A)
**Intensity of ADHD symptoms** Inattention Hyperactivity Total		19.83 (4.63) 16.14 (5.85) 35.83 (8.42)	
	**n (%)**		
**Gender** Male Female	11 (37.9%) 18 (62.1%)	51 (73.9%) 18 (26.1%)	0.001 (C)*
**ADHD subtype** Inattentive Combined Hyperactive-Impulsive		24 (34.8%) 42 (60.9%) 3 (4.3%)	
**DESR** Severe dysregulation Mild-moderate dysregulation Adequate emotional regulation		12 (17.4%) 40 (58.0%) 17 (24.6%)	

N = 98. (A): Mann-Whitney U test; (B) Student’s t-test; (C): Chi-square test (χ²); Significance level: <0.05. *Significant p-values.

The psychometric questionnaires showed no differences in anxiety symptoms between groups. Regarding the quality of life, differences were found in the domains of Risk avoidance (*p* < 0.001), which refers to risky behaviors that can affect the child’s health, and Achievement (*p* < 0.001), which includes expected functions for the respective age, i.e. performance in school and with peers. In both cases, the scores indicated a lower quality of life perception in the ADHD group.

Concerning the disorder, the ADHD subtype was mainly combined (60.9%), the mean intensity of symptoms score was 36 points, and most of the children (58%) presented mild to moderate DESR. Regarding comorbidities, 19% of the ADHD children presented generalized anxiety disorder, 10% had a social phobia, and 15% had oppositional defiant disorder (ODD).

### 3.1 Basal physiological parameters

The available physiological data of the ADHD group was compromised by movement artifacts. Due to the poor quality of the PPG signal, we will report only parameters extracted from the ECG. Firstly, we compared the raw average values between the TD group and the ADHD group at the Baseline stage (see **Table 3**). At this point, no data imputation was made.

**Table 3:** Comparison of physiological parameters between groups at Baseline.

Physiological parameter	Baseline stage				*p*-value (test)
	n	TD group	n	ADHD group	
HR	28	91.19 (8.88)	53	86.93 (11.23)	0.087 (A)
HRV					
SDNN RMSSD PLF PHF LF/HF PHFn	28 28 28 28 28 28	0.053 (0.02) 0.022 (0.01) 0.488 (0.30) 0.462 (0.35) 158.28 (128.76) 47.06 (12.39)	53 53 53 53 53 53	0.072 (0.04) 0.030 (0.02) 0.611 (0.34) 0.663 (0.63) 163.66 (109.99) 47.77 (14.91)	0.029 (B)* 0.124 (B) 0.071 (B) 0.316 (B) 0.796 (B) 0.832 (A)
PHFex PHFexn LF/HFex	28 28 28	0.581 (0.41) 54.36 (11.64) 98.72 (46.24)	53 53 53	0.850 (0.83) 54.43 (14.04) 107.76 (59.97)	0.311 (B) 0.984 (A) 0.684 (A)
PAT					
PAT stdPAT	18 18	203.68 (17.18) 7.12 (3.16)	10 10	211.44 (12.78) 8.43 (3.48)	0.224 (A) 0.472 (B)
Resp					
FR Pk	19 19	0.36 (0.08) 70.06 (3.65)	26 26	0.32 (0.09) 69.95 (2.47)	0.117 (A) 0.945 (B)
EDA					
mTonic stdTonic mPhasic stdPhasic aucPhasic EDASymp	29 29 29 29 29 29	-0.139 (0.64) 0.155 (0.10) 0.115 (0.13) 0.084 (0.06) 6.89 (7.57) 0.534 (0.80)	51 51 51 51 51 51	-0.530 (0.59) 0.240 (0.11) 0.074 (0.08) 0.067 (0.06) 4.46 (4.79) 0.316 (0.45)	0.004 (B)* <0.001 (B)* 0.041 (B)* 0.124 (B) 0.041 (B)* 0.198 (B)
ST					
TFace TGradFace TPowFace TFinger TGradFinger TPowFinger TRatio	29 29 29 29 29 29 29	30.77 (1.05) 0.009 (0.01) 948.05 (65.58) 28.67 (5.09) 0.023 (0.06) 847.25 (282.23) 0.933 (0.17)	65 65 65 31 31 31 27	33.07 (1.12) 0.004 (0.02) 1095.12 (72.86) 30.66 (5.28) 0.029 (0.10) 967.37 (310.33) 0.919 (0.16)	<0.001 (B)* 0.019 (B)* <0.001 (B)* 0.006 (B)* 0.750 (B) 0.006 (B)* 0.594 (B)

Mean (SD). n: available data included in the analysis. (A): Student’s t-test; (B): Mann-Whitney U test. Significance level: <0.05. *Significant p-values. No imputation was made.

Significant differences were found in some HRV, EDA, and ST parameters. The SDNN was higher in the ADHD group compared to the TD children (*p* = 0.029). Regarding the EDA, both the tonic (mTonic) and the phasic (mFasic) components were reduced in the ADHD group (*p* = 0.004 and *p* = 0.041, respectively). In contrast, the SD of the tonic component (stdTonic) was significantly higher in the ADHD group (*p* < 0.001). Both peripheral and central temperatures were higher in the ADHD group, and the average basal temperature was increased by 2.3°C with respect to the TD children.

### 3.2 Stress reactivity

**Figure 2:**
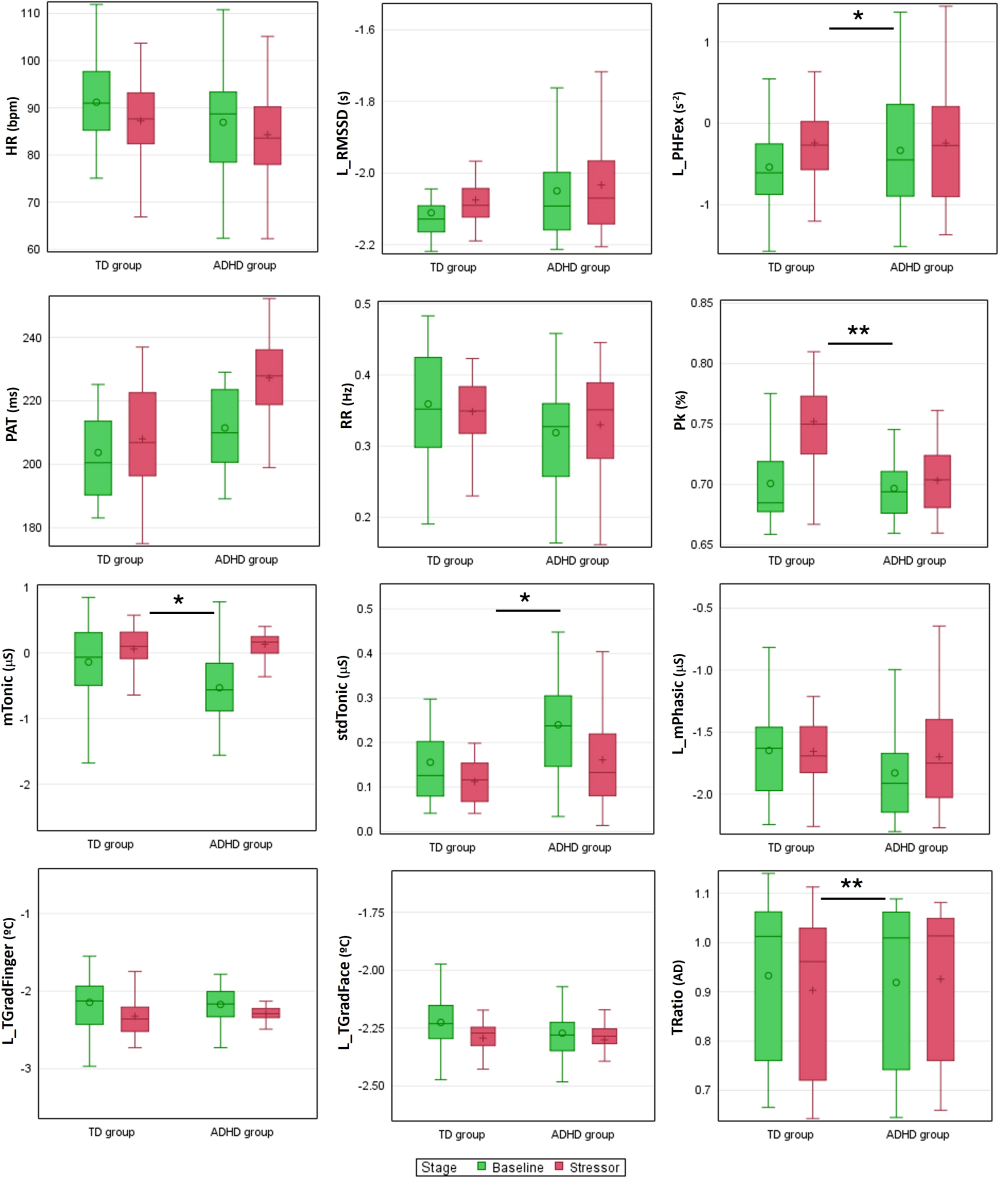
Boxplots of physiological parameters at Baseline and during the stress-inducing task. Differences in stress reactivity are indicated by *p<0.05 and **p<0.005. Some parameters (indicated by an L) were previously logarithmically transformed for better graphical visualization. bpm, beats per minute; AD, adimensional.

To mitigate the influence of physiological variability between individuals, we compared the stress reactivity, i.e. the difference between the Stress and the Baseline stages, which reflects the physiological response provoked by the sustained attention task (see **Figure 2**).

Both groups presented a similar response for HR and RMSSD. However, the TD group presented an increased response of PHF and, consequently, a reduced LF/HF compared to the ADHD group (for all results refer to **Supplementary Table 1** in **Supplementary Material**). This increased reactivity was even more pronounced in the HRV parameters in the extended band, which adjusts the frequency bands according to the average heart rate.

Greater reactivity for PAT was observed in the ADHD group, although not significant. Regarding respiration, the ADHD group showed slightly higher reactivity for RR, showing an increased response to the stress-inducing task. Furthermore, Pk presented lower values for the ADHD group, which suggests less stable breathing in this group.

The tonic-level EDA and its SD showed a higher reactivity in the ADHD group when compared to the TD group. About the temperature, this analysis indicated differences only in peripheral temperature, with increased reactivity in the finger temperature of the ADHD children when facing the Stress stage. Furthermore, the TRatio also differs between the groups, with TD children presenting a reduction in the respective value, which indicates that the peripheral and proximal temperatures move away from each other, e.g. the face temperature increases while the finger temperature decreases.

### 3.3 Multiparametric model design

Due to the sample imbalance related to the class label and gender, the PSM technique was applied as a compensation strategy. The procedure matched a TD child with one or more children of the ADHD group with similar characteristics. A total of 27 pairs were formed by 27 control children and 49 ADHD children, conforming to a new dataset that was used to design the model. To verify whether the procedure was effective, a mixed model for 2 effects (gender and age) was used, obtaining non-significant results after the procedure (gender: 0.012 to 0.775, and age: 0.058 to 0.062), which indicates that these variables have no longer influence on the children’s class label.

To reduce the number of physiological parameters and avoid overfitting, those highly correlated with each other were removed using the VIF criterion (61). The logistic regression model considered the children’s class label (ADHD *vs.* TD) as the response variable and all selected baseline physiological parameters as explanatory variables. Subsequently, a backward stepwise approach was employed to select the predictor variables. At this point, we decided not to include parameters such as the PAT, which had few real observations (n=10), and the temperature-related parameters due to the basal differences found between groups. Additionally, the variables gender and age, although adjusted by the PSM procedure, were included in the final model due to their importance related to the prevalence and intensity of ADHD symptoms supported in the literature (3, 62, 63).

The final model (ADHDm) includes 4 physiological parameters and is adjusted by age and gender (see **Table 4**). The variables gender and age do not present statistical significance, which represents the correct adjustment of the sample using the PSM before its design. Some physiological parameters show no significance either, however, in conjunction they contribute to the discrimination capacity of the model.

**Table 4:** Parameters and coefficients of the final model (ADHDm).

Parameter	Coefficients	Standard error	Wald Chi-Cuadrado	Pr > ChiSq
**Intercept**	4.6315	3.7986	1.4866	0.2227
**Gender | Male**	0.4627	0.8897	0.2705	0.6030
**Age**	-0.5639	0.4145	1.8504	0.1737
**RMSSD (log)**	0.8023	0.5566	2.0779	0.1494
**Fr**	-0.7679	0.4231	3.2937	0.0695
**stdTonic (log)**	2.8265	0.8582	10.8476	0.0010
**mPhasic (log)**	-2.2015	0.7825	7.9153	0.0049

Significance level < 0.05.

**Figure 3:**
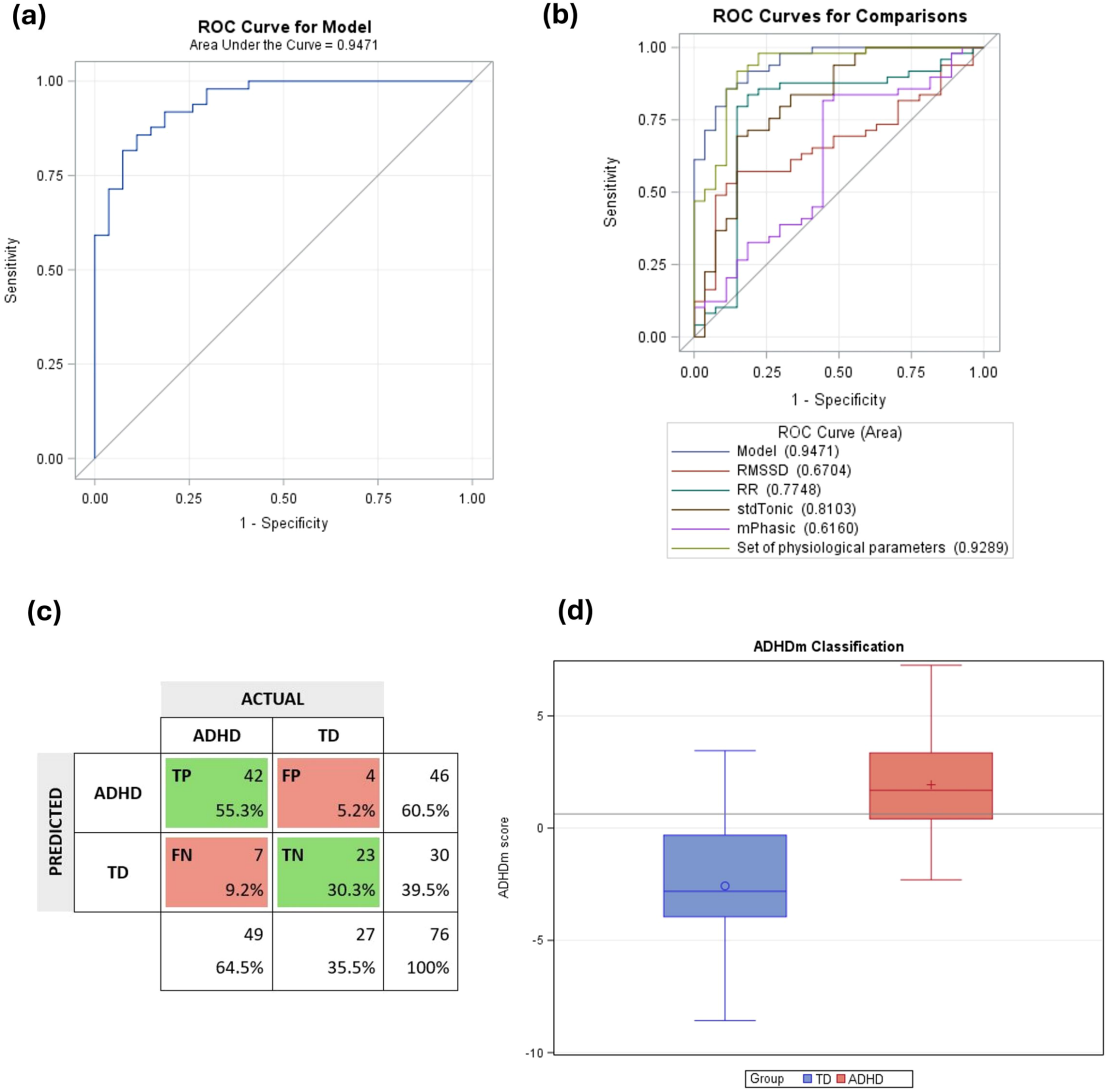
Performance of the ADHD classification model (ADHDm). Key: **(A)** ROC curve of ADHDm with an area under the curve of 0.95. **(B)** ROC curves for comparisons of physiological parameters’ contributions. **(C)** Confusion matrix. TP, true positives; FP, false positives; FN, false negatives; TN, true negatives. **(D)** Model’s classification for each group. ADHD, children with ADHD; TD, typically developing children. The darker gray line on the graph represents the cutoff point of 0.63.

The ADHDm obtained an area under the ROC curve of approximately 0.95, demonstrating the model’s good discrimination performance (**Figure 3A**). **Figure 3B** shows the ROC comparisons related to the physiological parameters included in the model. The RMSSD only presented an AUC of 0.67, the RR of 0.77, and the set of physiological parameters reached an AUC of 0.93, which supports the individual contribution of each one in the model characterization capacity.

To maximize the classification capacity of the model, the Youden index was used to select a cutoff point (64). This index proposes to find the probability point where the sum of sensitivity and specificity is greatest. For the studied sample, the sensitivity was 85.7% and the specificity was 85.2%, corresponding to the cutoff point of 0.63. An accuracy of 85.5%, a precision of 91.3% and a F1 score of 88.4% was obtained. **Figure 3C** shows the confusion matrix and **Figure 3D** shows the model’s classification according to the predicted ADHDm scores for each group.

We also performed an exploratory analysis to investigate whether the classification of intensity of symptoms (ADHD-RS-IV) and DESR, could influence the model’s performance. There is no significant effect regarding the DESR (*p* = 0.43) and the intensity of symptoms (*p* = 0.45), albeit the estimates are higher for the severe category (2.43 *vs.* 1.88 to mild and 1.68 to moderate categories).

We also investigated the effect of the ADHD subtype; similarly, no influence was found (*p* = 0.76). The estimates were higher for the inattentive subtype in comparison with the combined (2.04 *vs*. 1.87). In this instance, to ensure a more representative distribution of subtype categories, the three children diagnosed with the hyperactive-impulsive subtype were merged with those exhibiting the combined subtype.

## 4 Discussion

In this study, a set of basal physiological parameters was used to design a classification model of ADHD (ADHDm), which demonstrates a good discrimination capacity between ADHD and typically developing (TD) children. Limited research was found exploring physiological parameters in children with ADHD, most of the studies were centered on electroencephalogram (EEG) analysis (15). EEG is a more invasive and challenging method to apply in real-life context. In contrast, our multiparametric approach combines four physiological parameters that can be gathered with standard, non-invasive medical devices that are easily applicable in clinical settings.

The model’s adjustment by gender and age contributes to a more precise and personalized assessment, addressing key demographic factors that can influence physiological responses and the diagnosis of ADHD. Therefore, ADHDm represents an objective and accurate tool to support health professionals in the diagnosis process of ADHD. This model has the potential to complement traditional screening methods, which are often time-consuming and based on subjective clinical observations (12). By offering a quantitative approach, ADHDm could facilitate early intervention and improving patient outcomes. Moreover, this model aligns with the growing demand for non-invasive, real-world monitoring techniques in clinical practice. The ability to continuously monitor physiological parameters in real-time using wearable technology ensures a more personalized evaluation, which enables continuous observation of physiological dynamics and tailored interventions that address the unique needs of each child (65).

The basal physiological differences indicate greater HRV in ADHD children when compared with TD children. This finding is expected when the average heart rate is lower, which allows the heart rate variability to be greater (29, 66) but also consistent with abnormality in autonomic arousal present in ADHD (26). Both tonic and phasic components of the EDA signal were shown to be reduced in ADHD children, corroborating results from previous studies, indicating autonomic hypoarousal (28, 30–33). Negrao et al. (29) reported that the differences in EDA could be vanished by the use of stimulant psychotropic drugs. Similarly, Kim, Yang and Lee (67) found a reduction in HRV (RMSSD and PHF) after 12 weeks of stimulant treatment, suggesting that the predominance of the parasympathetic branch in ADHD can be modulated by medication. Given that ADHD children were drug naïve this influence can be discarded.

The stress reactivity analysis indicated that ADHD children may have a different response to stress compared to TD children. Regarding HRV, more notable changes were observed in TD children, with an increase in parasympathetic activity. On the contrary, tonic EDA showed a more prominent response in ADHD children, and also an increase in phasic EDA, although not significant. The peripheral temperature showed opposite patterns, increasing in ADHD children and reducing in the TD group. The TRatio was also affected, indicating a more pronounced stress response in TD controls. Additionally, an increment in the respiratory rate and lower respiratory peak were also observed in ADHD children, pointing to reduced breathing stability which is expected in children with hyperactivity.

The final logistic model showed a good capacity to discriminate ADHD children and TD controls, with an AUC of 0.95, an F1-score of 88.4%, an accuracy of 85.5%, a sensitivity of 85.7%, and a specificity of 85.2%. These results are equivalent to other approaches that applied machine learning techniques using demographic and clinical information (68), and cognitive task scores (69). Similarly, age and gender were commonly included in the models to improve their discrimination capacity (16). As an advantage, our model uses parameters that can be continuously measured by a wearable device in real-life scenarios. Thereby, through machine learning algorithms, the ADHDm could be reliably trained with labeled data and improve its estimation capacity.

### 4.1 Limitations

Although the sample size is small and further validation with larger samples is necessary, the results clearly indicate potential directions for refining and validating the model.

The signal recording was conducted with the same medical device; however, the time scale was different due to the different procedures adopted for recruitment, i.e. the hospital (for the ADHD group) and at the school (for TD children). In any case, all data collection occurred before the COVID-19 pandemic. Otherwise, there could be an important confounding factor, as the literature indicates increased mental health problems after the pandemic (70).

Another limitation was the imbalance between the class label (ADHD *vs.* TD) and gender. Whilst the same age range was set (7 to 12 years), the number of participants and the proportion of males and females could not be warranted owing to the unexpected interruption in the recruitment of TD children related to the COVID-19 pandemic. We intended to attenuate this imbalance with the PSM technique to reduce the bias when calculating the classification model. Although this strategy was used, it was finally decided to adjust the final model by gender and age due to their influence on ADHD disorder. The literature indicates a higher prevalence of ADHD in males, reaching a ratio of 3.3:1 for males and females respectively (62). The tendency to reduce the severity of symptoms with increasing age is also reported (63). Likewise, ADHD rating scales consider different scoring tables based on gender and age range.

Owing to logistical issues, the stress-inducing tasks applied in the experimental procedure were different for each group. While both tasks aim to evaluate the capacity for sustained attention, they are based on distinct paradigms. The CPT-3 (Conners Continuous Performance Task 3rd edition) (40), used for the ADHD group, is a 14-minute computerized task based on the continuous performance paradigm. In this task, participants must respond most of the time and inhibit certain stimuli (e.g. pressing the spacebar for any letter except X). It represents a Go-No Go task in which the attentional component is mixed with the capacity of motor inhibition.

Conversely, the CSAT-R (Sustained Attention Task in Infancy Revised) (41), used for the TD group, is a computer-based task grounded in the vigilance paradigm. In this task, participants are required to sustain attention and respond only to a specific stimulus (e.g. pressing the spacebar only if a 3-6 sequence appears). The surveillance mechanism activated by the CSAT-R may differ from that of the CPT-3, as reported by Servera, Sáez & Rodríguez (71). Consequently, the corresponding physiological data was examined solely to shed light on stress reactivity dynamics. However, the results should be interpreted with caution, as they do not permit firm conclusions.

### 4.2 Future research

We highlight some critical points to enhance replication and guide future research. First, standardizing tasks across different groups is essential to minimize variability in results and enable more robust conclusions regarding stress reactivity. Ensuring a balanced sample in terms of age, gender and body mass index (BMI) is equally important to avoid biases (72). Incorporating participants from diverse backgrounds and socioeconomic conditions will further generalizability of the findings (73).

Furthermore, including only treatment-naïve children allows for the clearest assessment of physiological patterns associated with ADHD. When children are on medication, its potential moderating effect should be carefully considered. The same applies to comorbid conditions, such as depression and oppositional defiant disorder (ODD), since the emotional self-regulation profile plays a crucial role in determining the most effective ADHD treatment (74).

Moreover, future investigations should focus on integrating wearable technology and machine learning algorithms to continuously monitor physiological parameters in real-world environments (36, 75). This approach would facilitate ongoing refinement of the ADHD model (ADHDm) by training it with larger datasets, enhancing predictive accuracy across diverse populations. Expanding the model to incorporate additional cognitive and executive function indexes could further improve its diagnostic accuracy, although this may limit its usability in real-time contexts. Alternatively, integrating measures related to activity level, motion, and ecological momentary assessments in longitudinal studies could provide deeper insights into disorder’s progression, daily behavior, treatment effects, and facilitate more personalized interventions for children with ADHD (76, 77).

## 5 Conclusions

Our study revealed increased parasympathetic (higher HRV) and reduced sympathetic (lower EDA) activation in children with ADHD, reinforcing the hypothesis of autonomic dysregulation associated with the disorder. The designed classification model combining a set of physiological parameters obtained a good discrimination power between ADHD and typically developing children. Therefore, it represents an objective and accurate tool to support health professionals in identifying children with ADHD and allowing the implementation of treatment at earlier stages. Further research is needed to explore larger datasets, fine-tune the model to better discriminate the severity of symptoms, and confirm the generalizability.

## Data Availability

The raw data supporting the conclusions of this article will be made available by the authors, without undue reservation.
