# Supplementary material for: Physiological parameters to support attention deficit hyperactivity disorder diagnosis in children: a multiparametric approach
Source: Front Psychiatry. 2024 Nov 7;15:1430797. doi: 10.3389/fpsyt.2024.1430797 (PMC11578978; doi:10.3389/fpsyt.2024.1430797)
Supplement: Supplementary file 1 [file Table1.docx]

Supplementary Material

The **Supplementary Table 1** shows the results of each physiological parameter regarding stress reactivity (i.e. the difference between Stressor and Baseline stages), to investigate how ADHD and typically developing (TD) children react to a sustained attention task.

**Supplementary Table 1.** Comparison of stress reactivity values between groups

| **Physiological reactivity** | **Δ Stress - Baseline** | | | | ***p*-value (test)** |
| --- | --- | --- | --- | --- | --- |
|  | **n** | **TD group** | **n** | **ADHD group** |  |
| HR | 28 | -3.954 (3.59) | 50 | -2.933 (4.04) | 0.293 (B) |
| **HRV** |  |  |  |  |  |
| SDNN | 28 | 0.006 (0.01) | 50 | 0.010 (0.02) | 0.080 (B) |
| RMSSD | 28 | 0.004 (0.003) | 50 | 0.004 (0.009) | 0.136 (B) |
| PLF | 28 | -0.027 (0.23) | 50 | 0.087 (0.29) | 0.138 (A) |
| PHF | 28 | 0.207 (0.21) | 50 | 0.066 (0.39) | 0.059 (B) |
| LF/HF | 28 | -68.60 (119) | 50 | 4.56 (118.3) | 0.005 (B)* |
| PHFn | 28 | 13.25 (10.01) | 50 | -1.83 (11.39) | < 0.001 (A)* |
| PHFex | 28 | 0.216 (0.22) | 50 | 0.066 (0.43) | 0.033 (B)* |
| PHFexn | 28 | 10.59 (8.26) | 50 | 0.85 (11.44) | <0.001 (A)* |
| LF/HFex | 28 | -33.23 (30.67) | 50 | -5.11 (51.65) | 0.004 (B)* |
| **PAT** |  |  |  |  |  |
| PAT | 18 | 5.21 (10.43) | 8 | 9.62 (9.01) | 0.657 (B) |
| stdPAT | 18 | -0.939 (1.27) | 8 | -0.066 (6.72) | 0.586 (B) |
| **Resp** |  |  |  |  |  |
| FR | 19 | -0.009 (0.05) | 25 | 0.013 (0.06) | 0.337 (B) |
| Pk | 19 | 6.11 (3.88) | 25 | 1.09 (2.80) | <0.001 (A)* |
| **EDA** |  |  |  |  |  |
| mTonic | 29 | 0.201 (0.79) | 61 | 0.664 (0.70) | 0.008 (A)* |
| stdTonic | 29 | -0.043 (0.10) | 61 | -0.079 (0.14) | 0.035 (B)* |
| mPhasic | 29 | -0.013 (0.09) | 61 | 0.025 (0.06) | 0.102 (B) |
| stdPhasic | 29 | -0.010 (0.06) | 61 | 0.023 (0.08) | 0.065 (B) |
| aucPhasic | 29 | -0.794 (5.37) | 61 | 1.488 (3.61) | 0.098 (B) |
| EDASymp | 29 | -0.166 (0.73) | 61 | 0.068 (0.26) | 0.178 (B) |
| **ST** |  |  |  |  |  |
| TFace | 29 | 0.205 (0.24) | 64 | 0.254 (0.36) | 0.703 (B) |
| TGradFace | 29 | -0.007 (0.01) | 64 | -0.004 (0.02) | 0.106 (B) |
| TPowFace | 29 | 12.58 (15.14) | 64 | 17.13 (24.43) | 0.562 (B) |
| TFinger | 29 | -0.705 (0.94) | 30 | 0.257 (1.39) | 0.001 (B)* |
| TGradFinger | 29 | -0.026 (0.08) | 30 | -0.031 (0.10) | 0.671 (B) |
| TPowFinger | 29 | -40.86 (55.76) | 30 | 12.31 (82.15) | 0.001 (B)* |
| TRatio | 29 | -0.030 (0.03) | 24 | -0.005 (0.03) | 0.004 (B)* |

Key: Mean (SD). N: available data included in the analysis. (A): Student's t-test; (B): Mann-Whitney U test. Significance level: <0.05. *Significant *p*-values. No imputation was made.
